# The influence of Bentonite content on the properties of its mixture with Kaolinite

**DOI:** 10.1038/s41598-025-89001-4

**Published:** 2025-03-31

**Authors:** Mohamed M. Salah, Mahmoud S. Hammad, Ayman L. Fayed, Ahmed M. Ebid

**Affiliations:** 1 Structural Engineering Department, Faculty of Engineering, Ain Shams University, Cairo, Egypt; 2 Faculty of Engineering and Technology, Future University in Egypt, New Cairo, 11865 Egypt

**Keywords:** Clay mixture, Index properties, Consistency limits, Compaction properties, Free swelling, Civil engineering, Mechanical properties, Mineralogy

## Abstract

This study investigates the index properties of artificially prepared mixtures of Bentonite and Kaolinite to simulate different types of fine-grained soils with diverse expansive characteristics, with a particular focus on understanding how various index characteristics influence swelling behavior. A series of controlled laboratory tests were conducted to evaluate the specific unit weight, free swell, consistency limits, and compaction parameters. The results show that altering the Bentonite content significantly affects the index properties of the mixtures, which in turn influences their swelling behavior. As the Bentonite content increases, the specific unit weight also increases due to the dense and compact nature of Bentonite. Additionally, the swelling properties of Bentonite cause substantial increases in the free swell and Atterberg limits, highlighting its critical role in controlling swelling potential and plasticity. Conversely, the maximum dry unit weight decreases with increasing Bentonite content due to its high water absorption capacity, which reduces compaction efficiency. These diverse index characteristics, such as free swell, Atterberg limits, and specific unit weight, are found to have a significant impact on the swelling behavior of the mixtures. By adjusting the Bentonite content, these mixtures effectively simulate a range of fine-grained soils with varying swelling potentials. These findings provide valuable insights into the behavior of soils with expansive properties, offering a foundation for optimizing their use in applications like engineered barriers, liners, and foundations, where controlling swelling and plasticity is essential.

## Introduction

Bentonite and Kaolinite are clay minerals that exhibit distinct behavior in response to water, primarily due to variations in their crystalline structures. Bentonite, predominantly composed of Montmorillonite, has a high water absorption capacity, leading to notable volumetric increase upon hydration. This volume increase is a result of water molecules entering the interlayer spaces of Montmorillonite, causing the mineral layers to separate, and resulting in significant swelling. In contrast, kaolinite, primarily composed of kaolinite minerals, behaves differently under hydration. While kaolinite can absorb water, it does not exhibit substantial volumetric expansion, attributed to its stable and tightly bound crystal structure, which restricts water penetration between its layers. Thus, the key distinction between these minerals is their response to hydration: Bentonite undergoes significant swelling due to interlayer water absorption, whereas Kaolinite exhibits minimal volume change due to its rigid structural configuration.

### Regarding consistency limits

The relation between the specific surface area (SSA) and the consistency limits of clays was studied using 267 test results from the literature, they concluded that the (SSA) alone is not enough to generate proper correlation and clay mineral types are essential in correlating the (SSA) with consistency limits^1^. In addition, an alternative testing procedure was presented to determine the liquid limit based on the required pressure to extrude the clay at a certain rate. The proposed test is more repetitive and less operator-dependent and its results match the ones from the falling cone test^2^. The critical role of surface area in influencing the behavior of fine-grained soils was highlighted by^3^, particularly concerning their geotechnical properties such as plasticity and moisture retention. While Atterberg’s Limits remain a widely accepted method for soil classification, they offer only a superficial understanding of the mechanisms driving soil behavior. According to^3^, soils with larger surface areas demonstrate a higher capacity for water absorption, resulting in increased plasticity and elevated liquid limits, characteristics commonly observed in expansive soils. Conversely, soils with smaller surface areas exhibit reduced water retention and lower plasticity^3^. emphasizes that measuring surface area provides a more comprehensive understanding of soil behavior, particularly in expansive or highly active clays, where traditional classification methods may fall short^4^ examined the relationship between the liquid limit, plastic limit, and clay content in various soils, demonstrating the ability to predict these limits based on clay content. For soils containing specific types of clay minerals^4^, established a direct correlation among these three factors, enabling accurate estimation of any one parameter when the other two are known. This finding provides a valuable framework for predicting soil behavior concerning its clay mineral composition^5–8^ in their study investigating Atterberg’s limits of the clay minerals Kaolinite, Illite, and Montmorillonite, as well as their respective mixtures with Sand, found that for moderate to high clay percentages, both the liquid limit and plastic limit are linearly correlated with the proportion of clay-sized particles in the samples.

The plastic limits for common pure clay minerals are detailed as follows according to^9^ and corroborated by^10^ the hierarchy of plastic limits is attapulgite superior to Montmorillonite, which is superior to Illite, which is superior to Kaolinite. For the liquid limits, the order is Sodium Montmorillonite superior to Calcium Montmorillonite, which is equivalent to attapulgite, followed by Illite and Kaolinite. Additionally, the plasticity index is ranked as sodium montmorillonite superior to calcium montmorillonite, which is superior to attapulgite, which is superior to Illite, which is superior to Kaolinite. The results also demonstrate that all Atterberg’s limits exhibit an increasing trend with decreasing particle size^11^ investigates the impact of liquid limit (LL) and cation exchange capacity (CEC) on swell percent (%S).A statistical model was developed to enable indirect estimation of %S based on LL and CEC. The multiple regression model demonstrated strong predictive performance, with a correlation coefficient (R² = 0.91), variance accounted for (VAF = 91.5%), and root mean square error (RMSE = 0.727). While CEC is a significant factor influencing the swelling behavior of clayey soils, a universally accepted quantitative classification for swelling potential does not currently exist. In this work, a new swelling potential classification system, comprising four zones—low, moderate, high, and very high swelling potential—was developed and proposed. This classification system provides engineers with a practical tool for evaluating soil expansively.

### Regarding compaction properties

Investigated^12,13^ the swelling behavior of three compacted soils with distinct physical properties under varying compaction energies, ranging from standard Proctor to modified Proctor compaction. To provide a broader comparison, they also studied Kaolinite and highly plastic Montmorillonite Clay. Their findings revealed that the percent swell (%S) is significantly influenced by the applied compaction energy. The percent swell (%S)–time relationship was observed to follow a rectangular hyperbola, enabling the prediction of the ultimate percent swell using a limited number of initial measurements. Furthermore, the swell–log time relationship exhibited three distinct phases: initial swelling, primary swelling, and secondary swelling. Notably, the secondary swelling phase was clearly distinguishable, and its rate provided valuable insights for predicting long-term swelling behavior. This study underscores the critical role of compaction energy in influencing the swelling characteristics of soils^14,15^. examined the factors governing the magnitude of volume change in compacted clays, identifying several key influences. These include the type and content of clay, the initial compaction conditions, and external factors such as the depositional environment. The depositional environment, in particular, plays a crucial role by affecting particle arrangement, overburden pressure, and the degree of weathering. Their findings highlight the interplay of these factors in determining the volume change behavior of compacted clays, offering a comprehensive understanding of the mechanisms involved.

### Regarding free swelling properties

Investigated^14,16^ the behavior of expansive clays in arid climates, where their development is significantly accelerated by desiccation and the weathering of shale. As urban expansion encroaches upon regions with swelling soils, the increased moisture content in these clays results in volume changes. These changes often lead to substantial damage to low-rise structures and pavements, highlighting the critical need for appropriate geotechnical considerations in construction and urban planning in such areas.

Explored^15^ the mechanisms underlying the volume increase in clay soils upon water exposure, attributing this behavior to the chemical composition of the soil and the mineral types present. Shahm emphasized that the phenomenon of volume change is not solely governed by the activity of clay particles themselves but is predominantly influenced by the surface activity of these particles and the spacing between them. This insight underscores the importance of particle interactions and surface chemistry in understanding the swelling and shrinkage behavior of clay soils.

Explored^17–20^ the relationship between swelling properties and more easily obtainable soil parameters by utilizing various Clay mineral mixtures to cover a broad range of plasticity indices. Recognizing the time-intensive nature and high cost of directly measuring swelling properties, the study aimed to identify simpler soil characteristics that correlate with swelling behavior. Through multiple regression analysis, the free swell percentage was correlated with variables such as clay content, water content, dry unit weight, plasticity index, liquidity index, and cation exchange capacity. This approach offers a more accessible method for predicting swelling behavior based on widely measurable soil properties.

### Regarding ML predictive models

Recently, machine learning techniques were extensively utilized to generate predictive models that correlated soil classification and physical properties with its mechanical and swelling properties. For example:

- Predicting swelling potential of clayey soils using the plasticity index and clay content^21^.
- Estimating undrained shear strength of clay using the density and consistency limits^22^.
- Calculating swelling stress of the expansive clay using its free swell index, matric suction and density^23,24^.
- Evaluated unconfined compressive strength of expansive clay using its activity, plastic index and shear parameters^25^.
- Finally, predicted the swelling of Egyptian shale considering the contents of nanoparticle content in the surrounding water^26^.

The previous review showed the huge efforts of the earlier researcher to study the behavior of clays (especially the swelling ones) and to correlate their properties using different mathematical and soft computing techniques. However, there is still an opportunity to conduct more research to fill the remaining gap studies. Accordingly, this research aims to correlate the classification, physical, compaction and swelling test results of (Bentonite-Kaolinite) mixture with the bentonite content. The developed formulas could be used as a scale to evaluate the behavior of different clay samples.

## Methodology

**Fig. 1:**
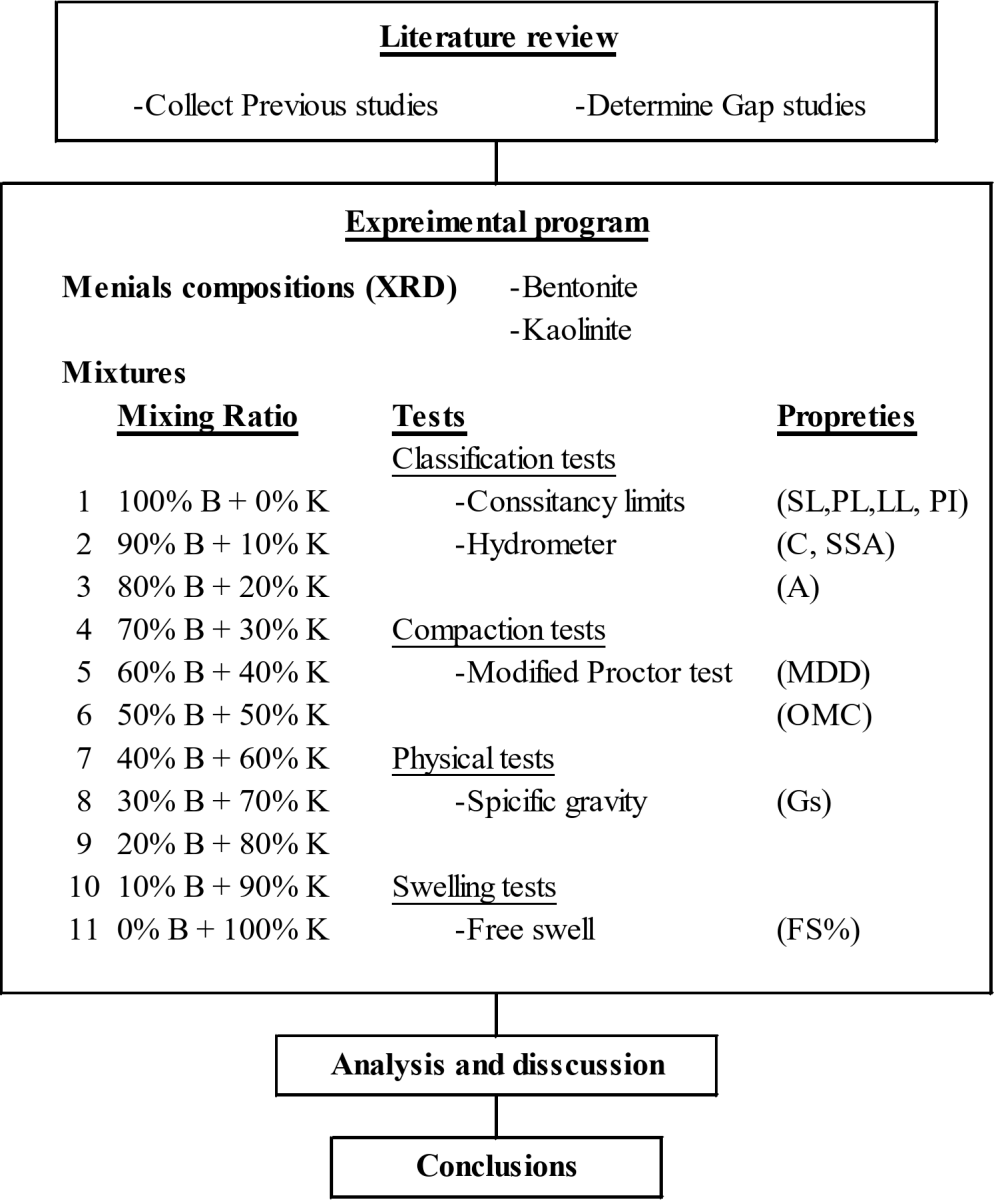
The considered methodology.

The considered methodology in this research began with collecting, sorting, studying and summarizing the previous related research works. Based on the determined gap study, the experimental research program was developed. This program was divided into two phases, the first was to study the considered clay minerals (Bentonite & Kaolinite) using the “X-ray deviation” (XRD) technique. The second phase was dedicated to determining the classifications, compaction, physical and swelling properties of mixtures of Bentonite and Kaolinite with different ratios. The results of the experimental program were analyzed and discussed to draw conclusions and recommendations. Figure 1 presents the considered methodology.

## Experimental research program

### Considered clay minerals composition using (XRD)

The samples used in this study, consisting of Kaolinite and Bentonite, were selected for their continuous application in geotechnical engineering. These materials were provided by reputable suppliers to ensure their quality and consistency. This procurement aimed to facilitate accurate and reproducible experimental results in line with the standards required for geotechnical engineering applications.

**Fig. 2:**
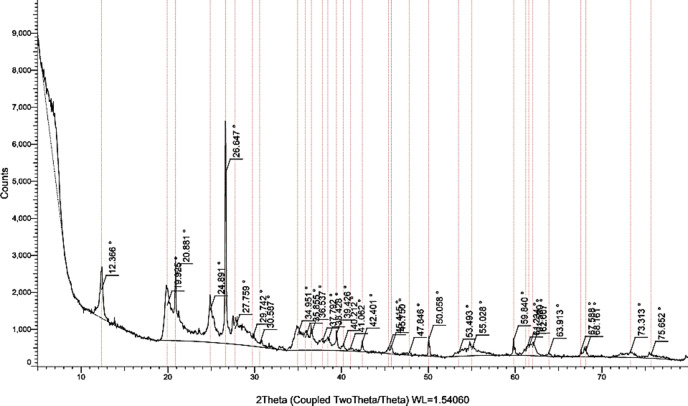
XRD test results for Bentonite sample.

To ensure that the provided samples were montmorillonite and kaolinite, X-ray diffraction (XRD) tests were conducted to characterize their mineralogical composition and crystalline structure. The XRD patterns of the Bentonite sample as shown in Fig. 2 revealed prominent peaks characteristic of montmorillonite, the primary mineral in bentonite, with diffraction peaks observed at specific 2θ values around 7° (001 plane) and 19° (002 plane), indicating a layered silicate structure. Additionally, the analysis showed the presence of other minor minerals, such as quartz and feldspar, which may influence the swelling behavior of Bentonite.

**Fig. 3:**
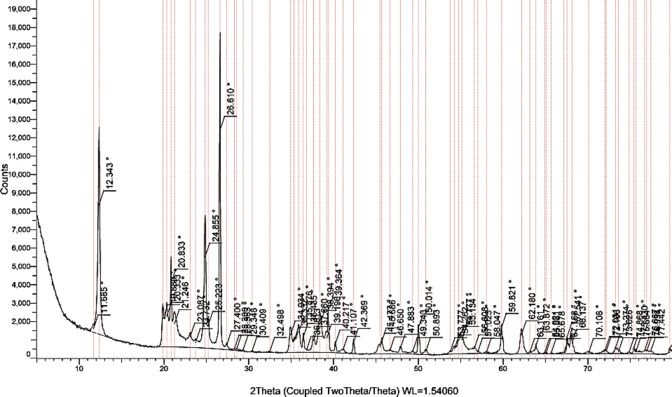
XRD test results for Kaolinite sample.

In contrast, the XRD results for Kaolinite as shown in Fig. 3 exhibited sharp and well-defined peaks, particularly at 12° (001 plane) and 24° (002 plane), confirming Kaolinite as the dominant mineral and indicating a relatively pure sample with the absence of significant impurities. The well-defined peaks signify the crystallinity of Kaolinite. A comparative analysis of the XRD patterns for both clays demonstrated distinct differences in their mineralogical compositions. While bentonite displayed a more complex structure with significant montmorillonite content, kaolinite was characterized by its relatively simple, crystalline structure.

This contrast in mineralogy is expected to have implications for the swelling behavior of the clay mixtures. The XRD analysis confirms that both Bentonite and Kaolinite are suitable candidates for investigating the influence of their mixtures on swelling behavior. The mineralogical composition of each clay will play a crucial role in determining the overall swelling potential of the mixtures, particularly given the expansive nature of montmorillonite found in bentonite. Overall, the XRD results provide essential insights into the structural characteristics of the Bentonite and Kaolinite samples, laying the groundwork for further analysis of their behavior in swelling tests and their application in geotechnical engineering.

### Bentonite and Kaolinite mixtures

A systematic approach was adopted to prepare mixtures of Bentonite and Kaolinite to investigate their effects on swelling behavior. Bentonite and Kaolinite were mixed by the following mixing technique:

1. Dry both Bentonite and Kaolinite powders to remove any moisture content that might affect mixing.
2. Sieve the powder using a 2 mm mesh to remove any larger particles or impurities.
3. Accurately weigh the required quantities of Bentonite and Kaolinite according to the desired proportions.
4. Place the weighed powders in a clean mixing container.
5. Mix the dry powders in a mixing drum at a speed range of 50 to 100 RPM. This low-speed mixing helps minimize dust generation while ensuring the even distribution of the powders and preventing material segregation, resulting in a homogeneous blend. Mix thoroughly for 5 to 10 min to achieve uniform distribution.
6. After the dry mixing process, a mixture of bentonite and kaolinite is formed, which can be used in various tests.

Various proportions of Bentonite and Kaolinite were prepared to create a series of mixtures, with Bentonite contents starting with 0%, 10%, 20%, and up to 100% and corresponding reductions in Kaolinite. The selected proportions were completely mixed using a mechanical mixer to achieve a homogenous mixture, ensuring that the two components were evenly distributed throughout the mixture.

The prepared mixtures were then subjected to standardized laboratory tests to measure swelling pressure and swelling potential, conducted under controlled temperature and humidity conditions to minimize external influences on the results. The results from the swelling tests were meticulously recorded, and data analysis involved correlating the bentonite content with the observed swelling behavior to identify trends and patterns. This methodology provides a structured framework for examining the influence of bentonite content on the swelling behavior of Kaolinite, facilitating a comprehensive understanding of the interactions between these materials in geotechnical applications.

### Tests procedure

This study investigates the properties of Kaolinite and Bentonite, which are commonly utilized in geotechnical applications. It also examines the effects of mixing these materials in varying proportions to create different mixtures and assesses the geotechnical properties of these mixtures. Six different laboratory tests were carried out for each mixing ratio. All the tests were conducted in the geotechnical research lab, faculty of engineering, Ain Shams University. The conducted tests and the extracted parameters during this study are listed in Table 1.

**Fig. 4:**
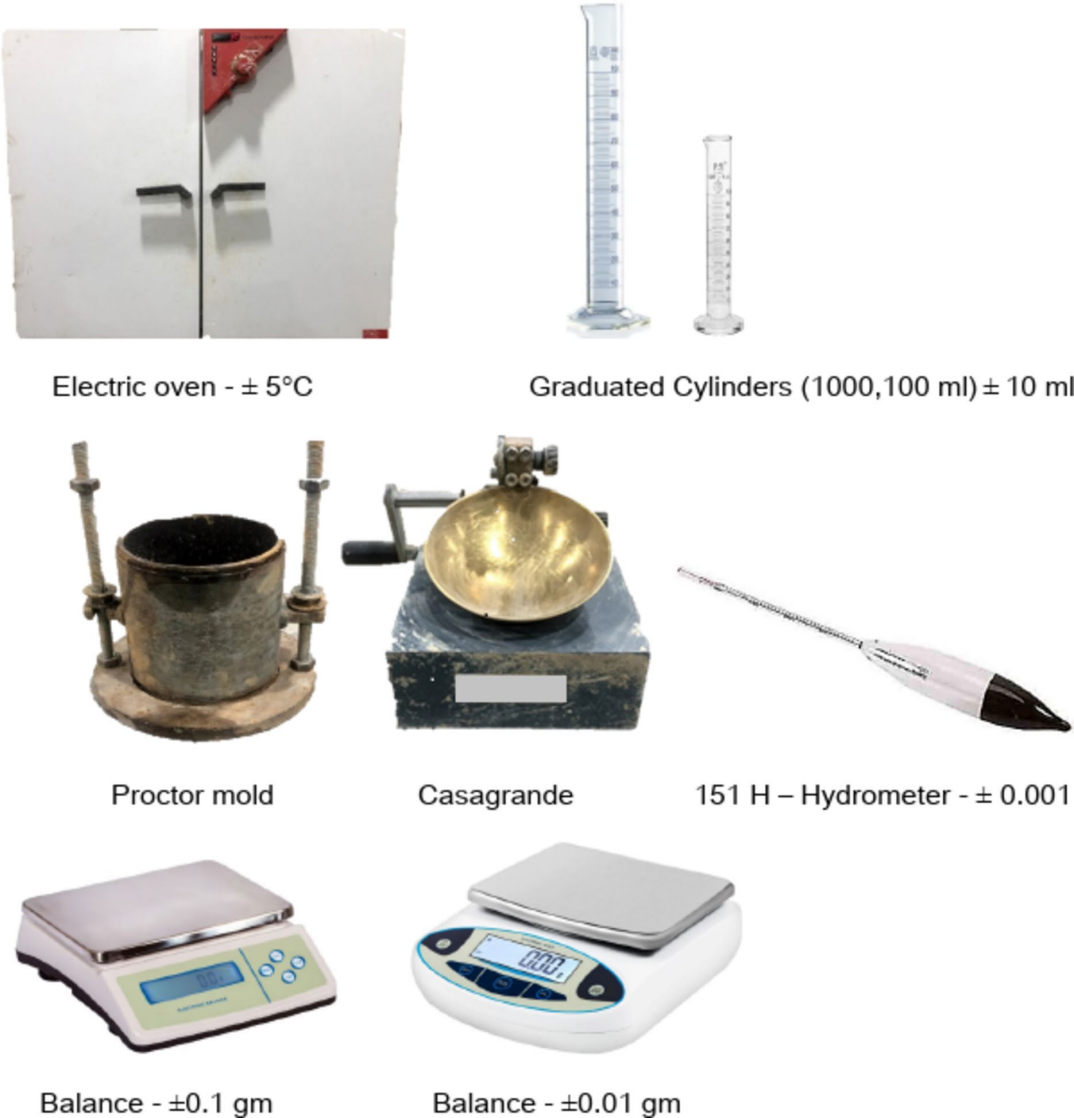
Devices and apparatus used in this study.

The devices and apparatus used in this research were chosen to evaluate the physical and geotechnical properties of Bentonite-Kaolinite mixtures effectively. The Casagrande apparatus was employed to determine the liquid limit of the mixtures, providing critical insights into their consistency limits. A Proctor mould was used to measure the maximum dry density and optimize the compaction characteristics of the samples. Sedimentation analysis was conducted using a graduated cylinder and a hydrometer to assess particle size distribution, highlighting the clay fraction in the mixtures. Additionally, a graduated cylinder was used to perform free swell tests, measuring the swell percent of the mixtures when exposed to water. These devices played a vital role in ensuring accurate and reproducible data throughout the study. The used devices and apparatus are presented in Fig. 4.

**Table 1:** The considered tests and their references.

Category	Test	Parameters	Reference
Classification	Consistency (or Atterberg) limits	Liquid limit (LL) Plastic Limit (PL) Plasticity Index (PI)	ASTM D4318-05^27^
	Hydrometer	Specific surface area (SSA) Clay content (C) Activity Index (A)	ASTM D7928-21^28^
Compaction	Modified Proctor	Max. dry density (MDD) Optimum moisture content (OMC)	ASTM D1557-12^29^
Physical	Specific gravity	Specific gravity (Gs)	ASTM D854-14^30^
Swelling	Free swelling	Free swelling percent (FS)	ASTM D5890-10^31^

## Test program results

**Fig. 5:**
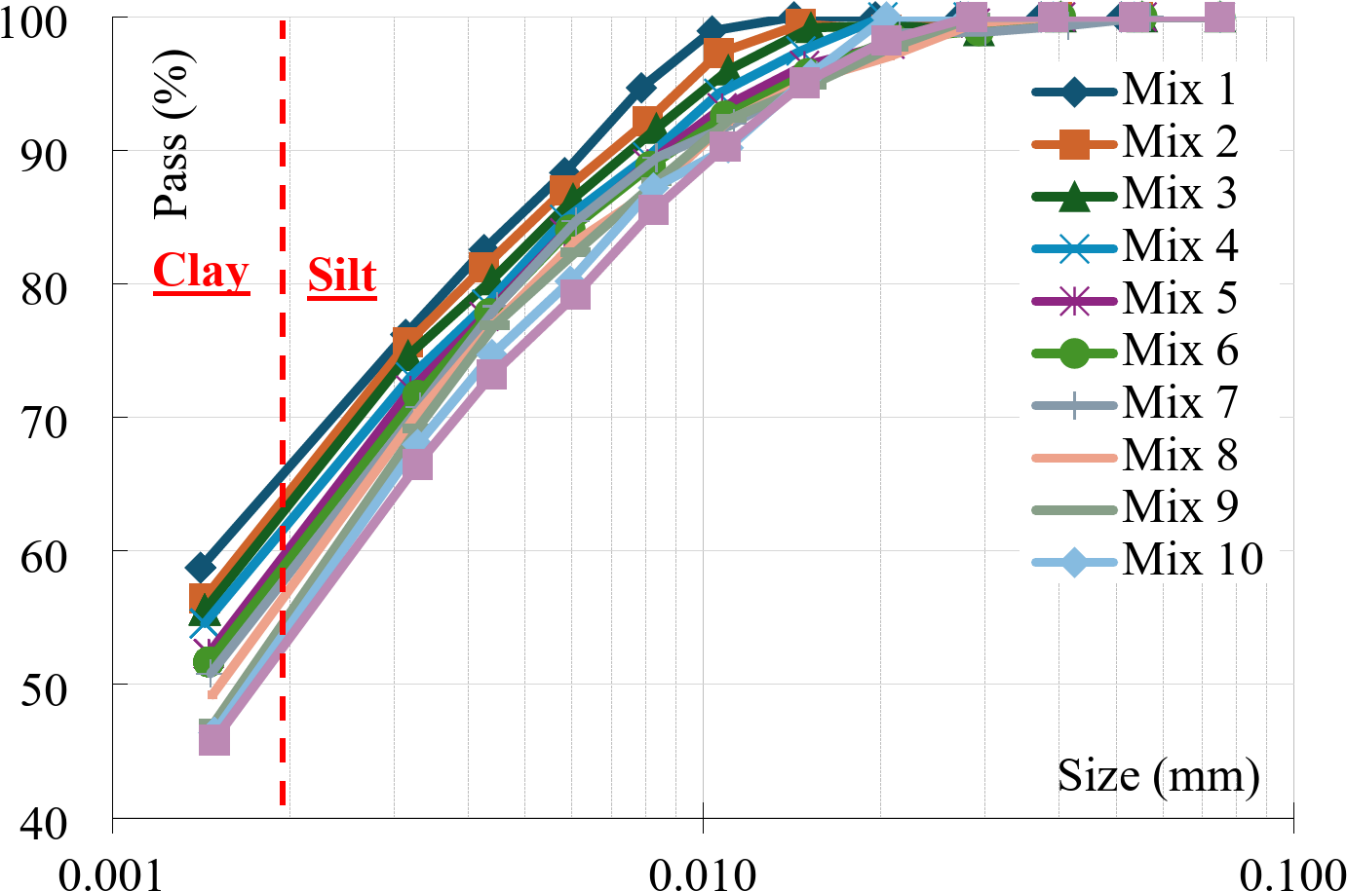
Grain size distribution curves for all mixtures.

**Fig. 6:**
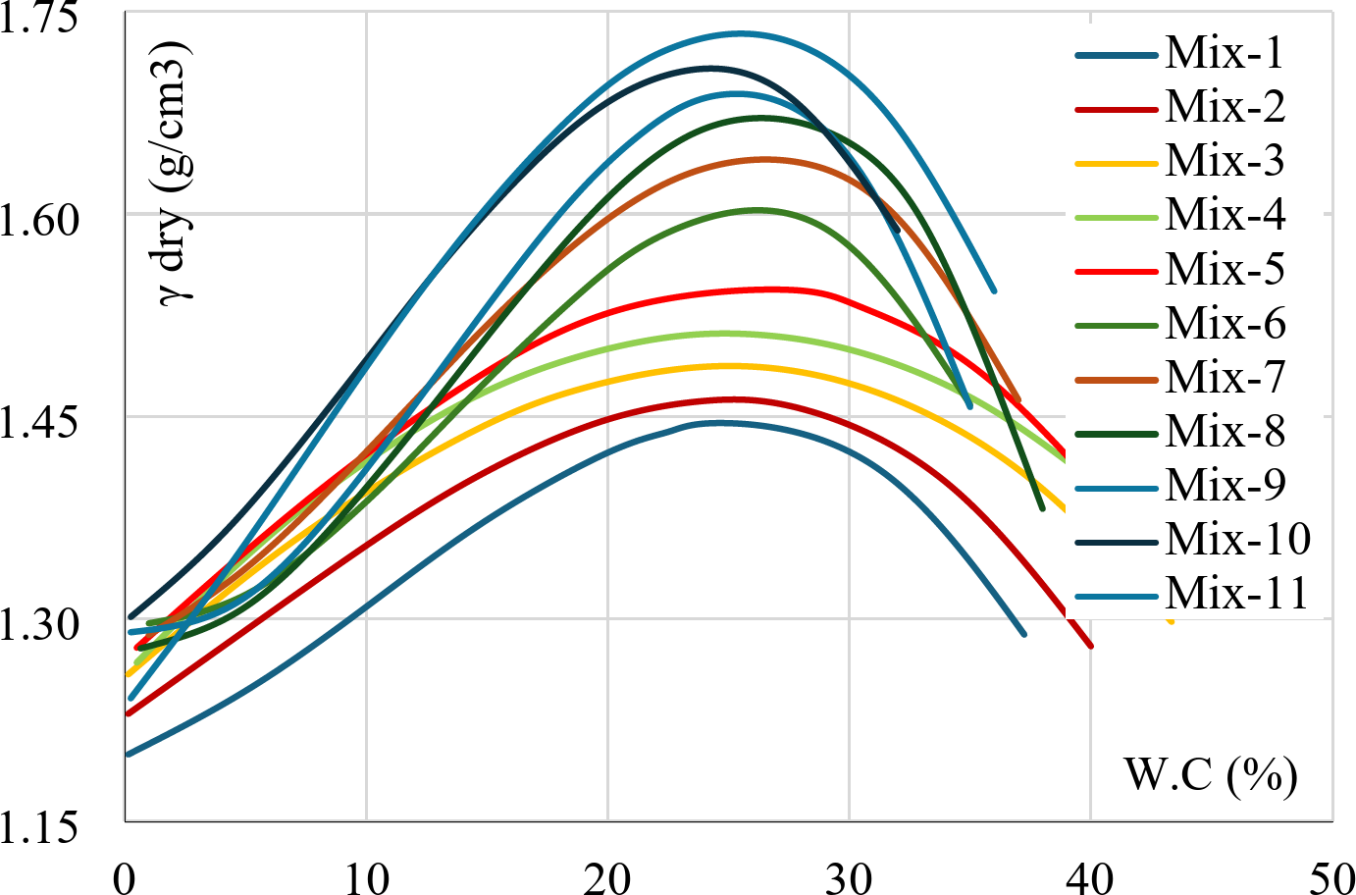
Compaction curves for all mixtures.

The results of the testing program for all eleven mixtures are summarized in Table 2. In addition, the results of the hydrometer tests of all the considered mixtures are graphically presented in Fig. 5, where (Mix 1) is pure Bentonite and (Mix 11) is pure Kaolinite. The figure showed that Bentonite is finer than Kaolinite. In other words, the Clay content (particles finer than 0.002 mm) in Bentonite is higher than in Kaolinite. Finally, Fig. 6 includes the results of all modified Proctor tests. The graph showed that the compaction curve of pure Kaolinite is the highest and the curve of pure bentonite is the lowest.

**Table 2:** Summary of the experimental program results.

Mix	B	K	S.L	*P*.L	L.L	*P*.I	C	SSA	A	Gs	MDD	OMC	FS
ID	(%)	(%)	(%)	(%)	(%)	(%)	(%)	(cm^2^/g)	(%)	-	(g/cm^3^)	(%)	(%)
1	100	0	12.7	52.6	239.5	186.9	66.4	369,787	2.82	2.74	1.45	25.3	730
2	90	10	12.9	48.2	210.9	162.7	66.3	360,812	2.45	2.72	1.46	24.9	660
3	80	20	12.5	39.7	170.5	130.8	63.6	357,391	2.06	2.70	1.49	25.0	470
4	70	30	13.3	38.1	148.6	110.5	62.8	352,758	1.76	2.68	1.51	25.8	370
5	60	40	13.8	35.8	128.8	93.0	60.8	348,468	1.53	2.66	1.55	25.8	300
6	50	50	13.5	30.6	104.7	74.1	58.6	342,617	1.26	2.65	1.60	26.1	250
7	40	60	13.7	28.0	89.2	61.2	57.9	339,278	1.06	2.63	1.64	26.4	180
8	30	70	13.8	25.5	74.6	49.1	56.4	337,965	0.87	2.61	1.67	25.8	90
9	20	80	14.3	22.60	55.9	33.3	55.3	335,078	0.60	2.59	1.69	25.2	40
10	10	90	16.0	21.3	39.8	18.5	54.8	333,712	0.34	2.57	1.71	24.1	10
11	0	100	17.7	20.4	28.9	8.5	54.3	330,988	0.16	2.55	1.75	22.8	0

## Analysis and discussion

### Clay content (C)

**Fig. 7:**
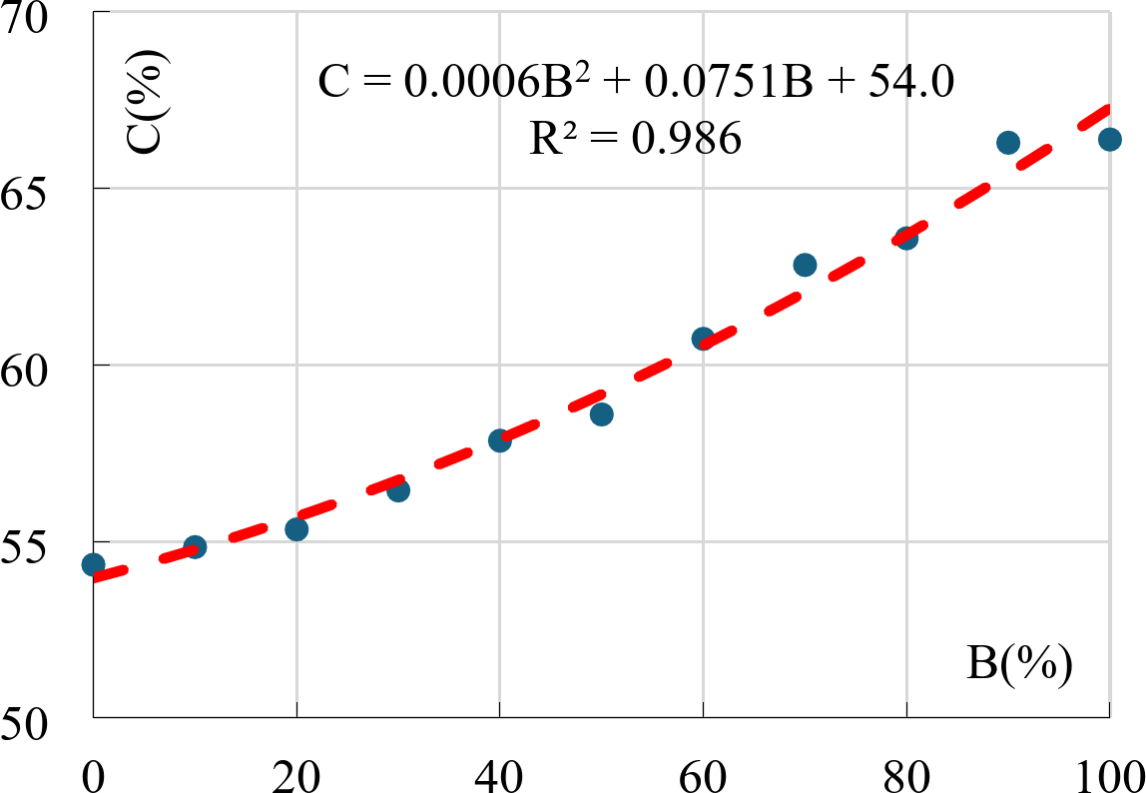
Clay content vs. Bentonite content.

Clay content (C) is defined as the percentage of the particles finer than 0.002 mm. Because the Bentonite particles are finer than the Kaolinite ones, the clay content (C) increased by increasing the Bentonite content (B). As shown in Table 2, the clay content varied between 54.3% at B = 0.0% (Mix 11) up to 66.4% at B = 100% (Mix 1). The relation between clay and bentonite contents is graphically presented in Fig. 7.

### Specific surface area (SSA)

**Fig. 8:**
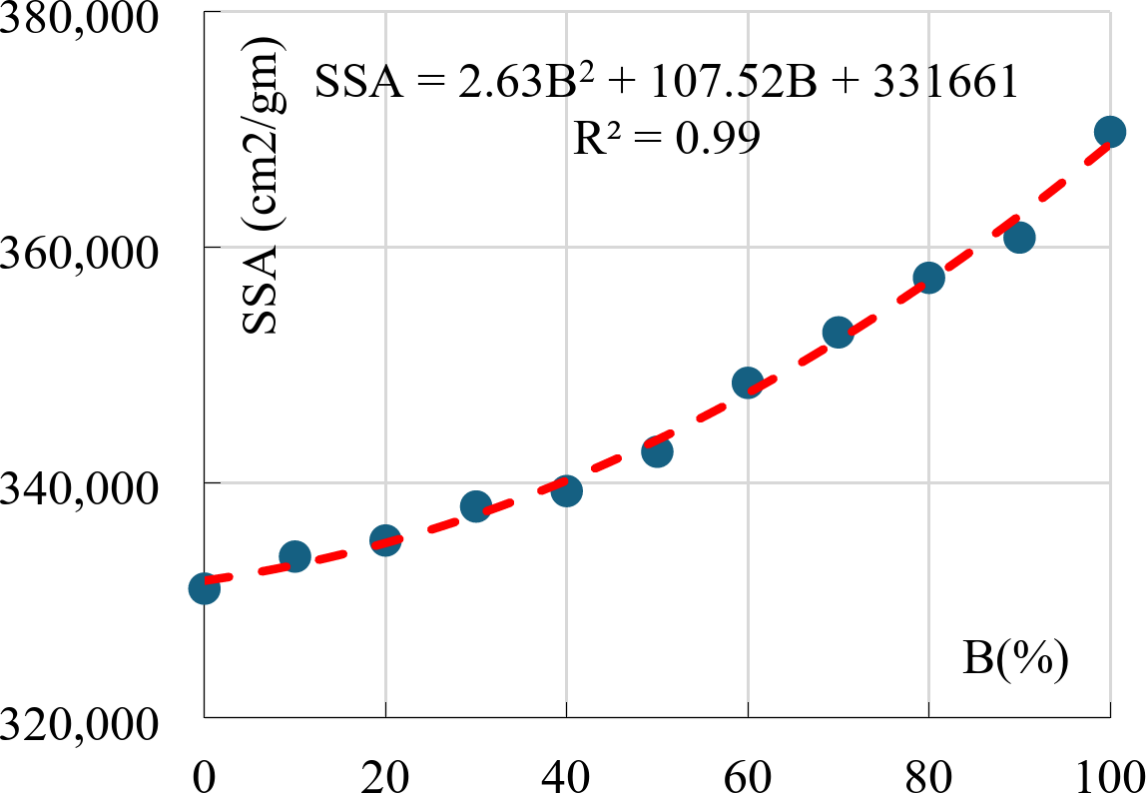
SSA vs. Bentonite content.

In the same context, the Specific surface area (SSA) of the mixture increased by increasing the Bentonite content. The SSA varied between 330,988 and 369,787 cm3/g for pure Kaolinite and pure Bentonite respectively, Fig. 8 shows the relation between SSA and Bentonite content.

### Consistency limits

**Fig. 9:**
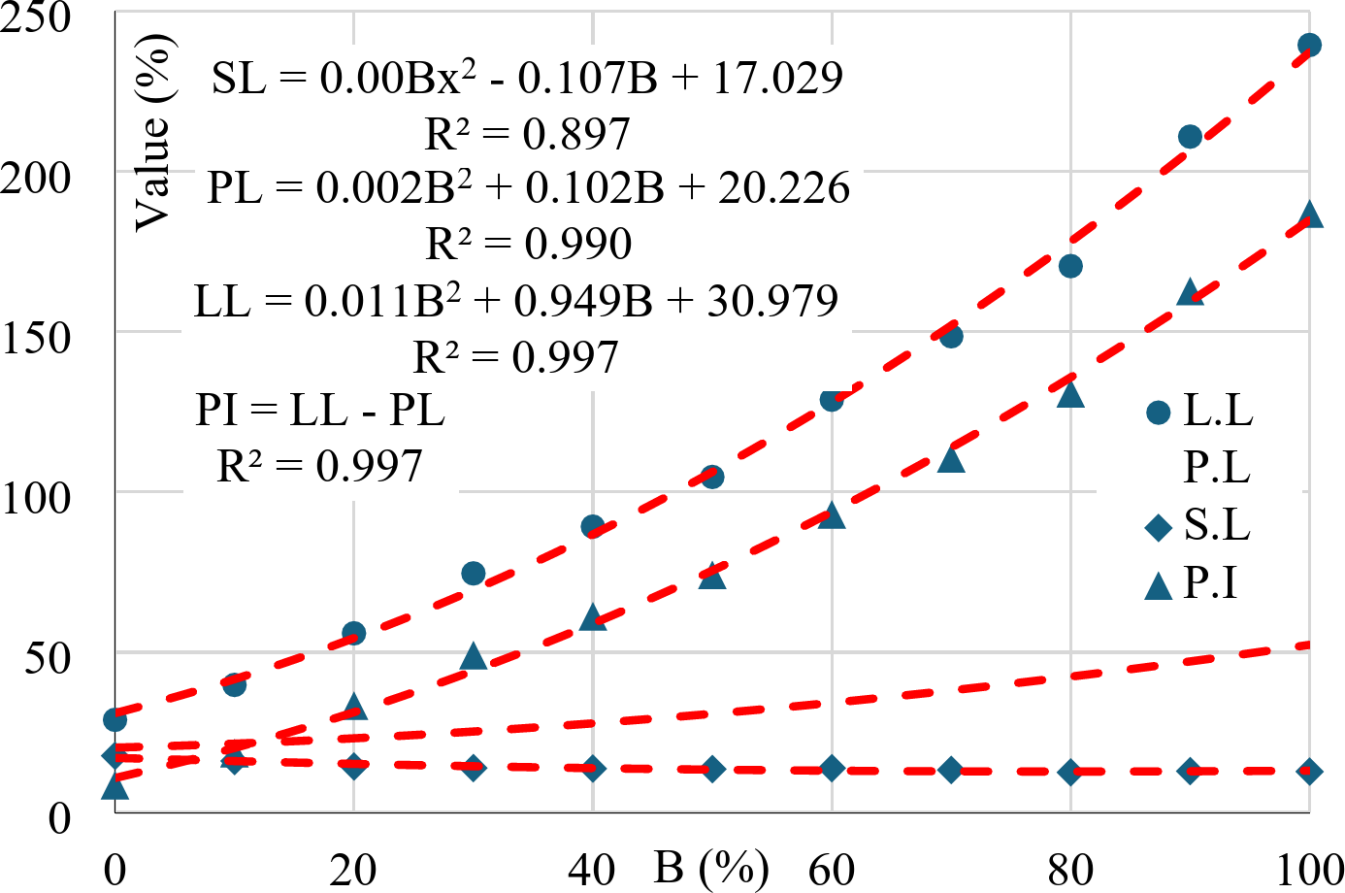
Consistency limits Vs. Bentonite content.

As listed in Table 2, all consistency limits increased by increasing the Bentonite content, but at different rates. The variation in (LL) was huge, about 830% (from 29 to 240% for pure Kaolinite and pure Bentonite respectively). The change in (PL) was moderate, about 260% (from 20 to 52% for pure Kaolinite and pure Bentonite respectively). Finally, the change in (SL) was limited, about 38% (from 13 to 18% for pure Kaolinite and pure Bentonite respectively). The relation between the three consistency limits (SL, PL & LL) and the Bentonite content in the eleven considered mixtures are graphically presented in Fig. 9. The graph indicated a non-linear relation between (LL) and Bentonite content, while the relation between (PL) and (B) was almost linear. Accordingly, the relation between (PI = LL-PL) and (B) was nonlinear and almost parallel to (LL) curve. Finally, (SL) value was almost constant (15.5%±2.5%). The huge ability of Bentonite to absorb water is the reason for the increase in both (PL) and (LL) of its mixtures.

### Activity index (A)

**Fig. 10:**
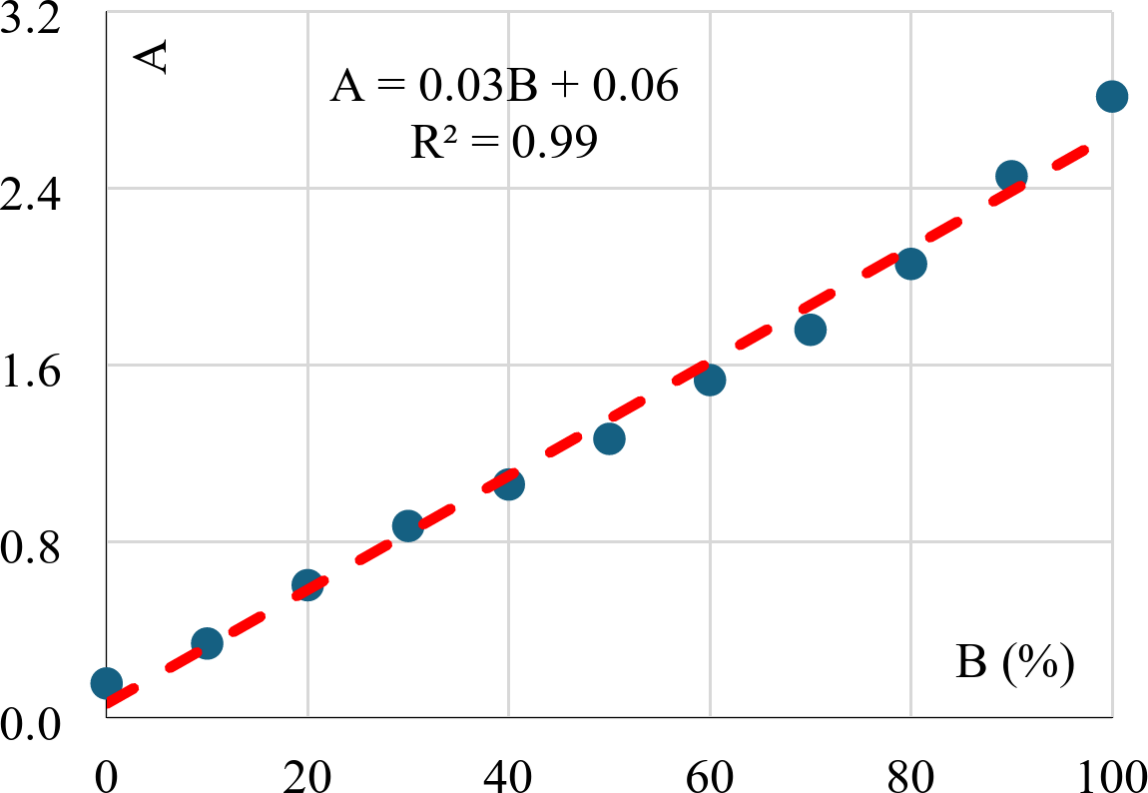
Activity index vs. bentonite content.

Clay activity is the ratio between (PI) and clay content (C). It is a good indicator of the swelling potential of the clay. The results indicated that the clay activity varies proportionally with bentonite content as shown in Fig. 10. This is reasonable since Kaolinite is non-swelling clay and Bentonite is a very high-swelling clay.

### Swelling potential (SP)

**Fig. 11:**
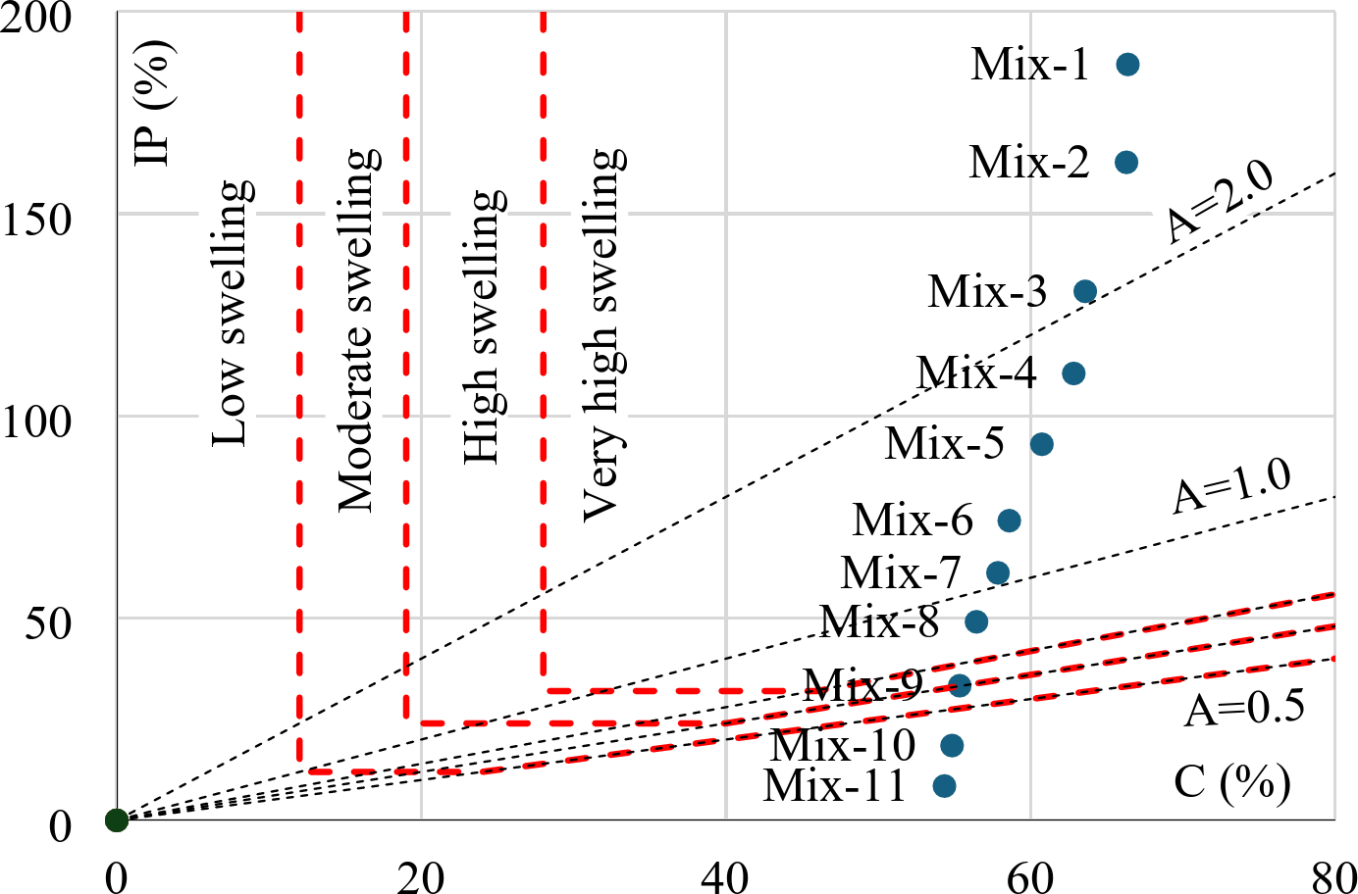
The swelling potential of the considered mixtures.

The swelling potential of certain clay could be determined based on its (PI) and (C) values. Figure 11 presents the swelling potential classification for the eleven considered mixtures. It indicated that mixtures with B < 15% are classified as low swelling, 15% < B < 20% as moderated swelling, 20% < B < 25% as high swelling and finally for B > 25% as very high swelling.

### Maximum dry density (MDD)

**Fig. 12:**
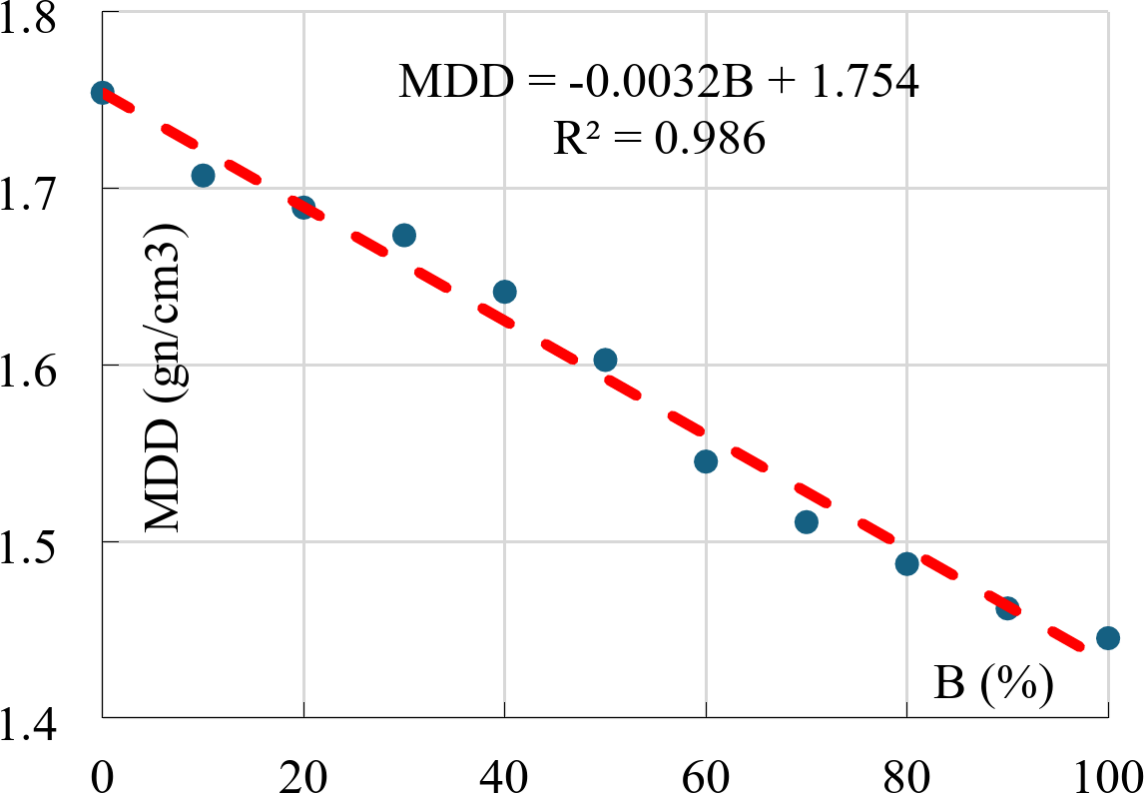
Maximum dry density vs. Bentonite content.

The results of modified Proctor tests showed that the (MDD) linearly decreases with increasing the Bentonite content as shown in Fig. 12. This decrease in density was due to the additional absorbed water by Bentonite, which increased in volume (swelling) and accordingly decreased in density.

### Optimum moisture content (OMC)

**Fig. 13:**
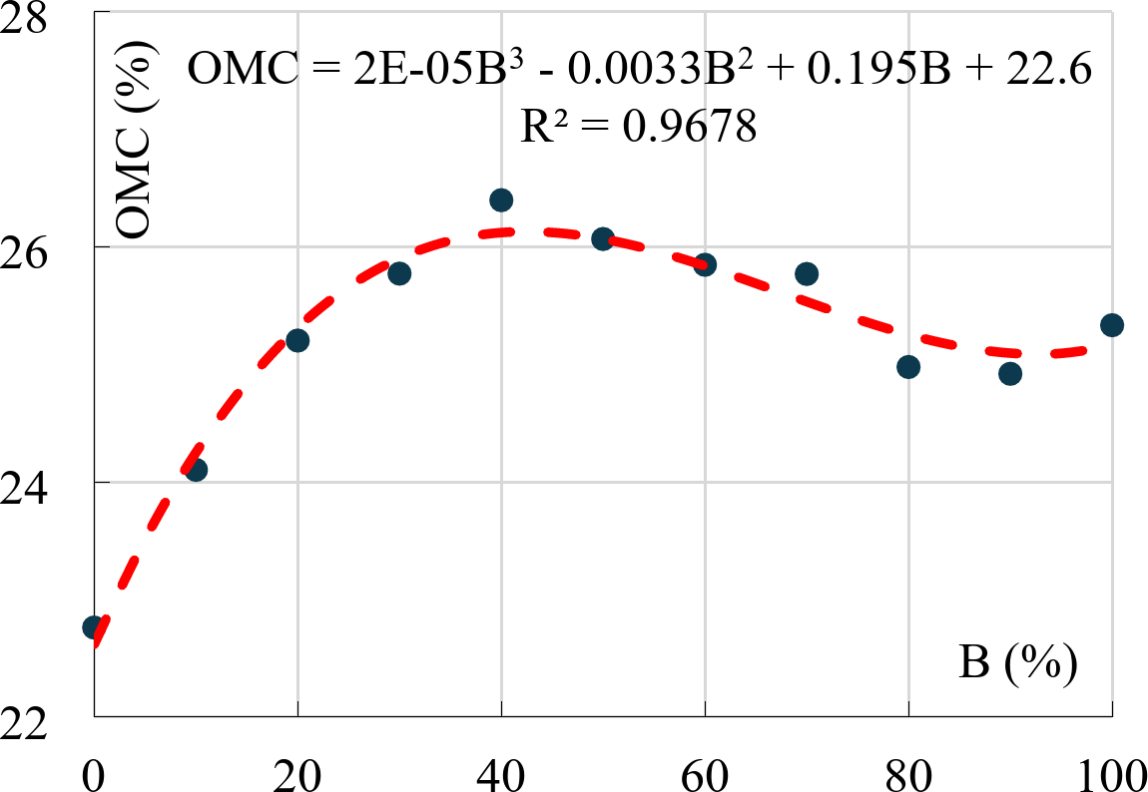
Optimum moisture content vs. Bentonite content.

In the same context, the modified Proctor tests showed that the relation between (OMC) and bentonite content is complicated, it begins linearly from 23% at B = 0–25% at B = 20%, above this content, it is almost constant at 25.5% ± 0.5% as shown in Fig. 13. Comparing the results from Figs. 12 and 13 showed that the reduction in (MDD) for low Bentonite mixtures (B < 20%) was due to the additional absorbed water, while the reduction in (MDD) beyond this limit was because of the increase in volume due to Bentonite swelling (at constant moisture content). This analysis matches the previously discussed swelling potentials.

### Specific unit weight (gs)

**Fig. 14:**
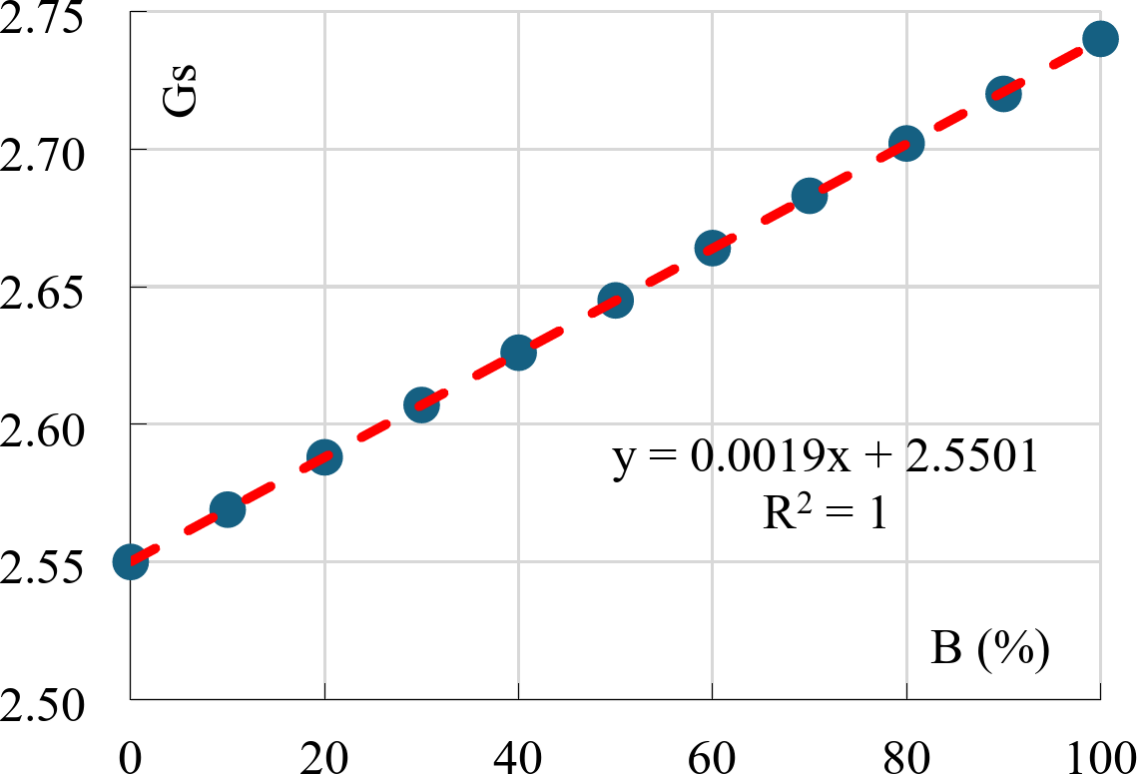
Specific unit weight vs. Bentonite content.

As expected when mixing two materials with different specific unit weights, the (Gs) value of the mixture is the weighted mean between the (Gs) values of the two pure materials. Figure 14 shows the linear relation between the (Gs) value of the mixture versus its Bentonite content.

### Free swell

**Fig. 15:**
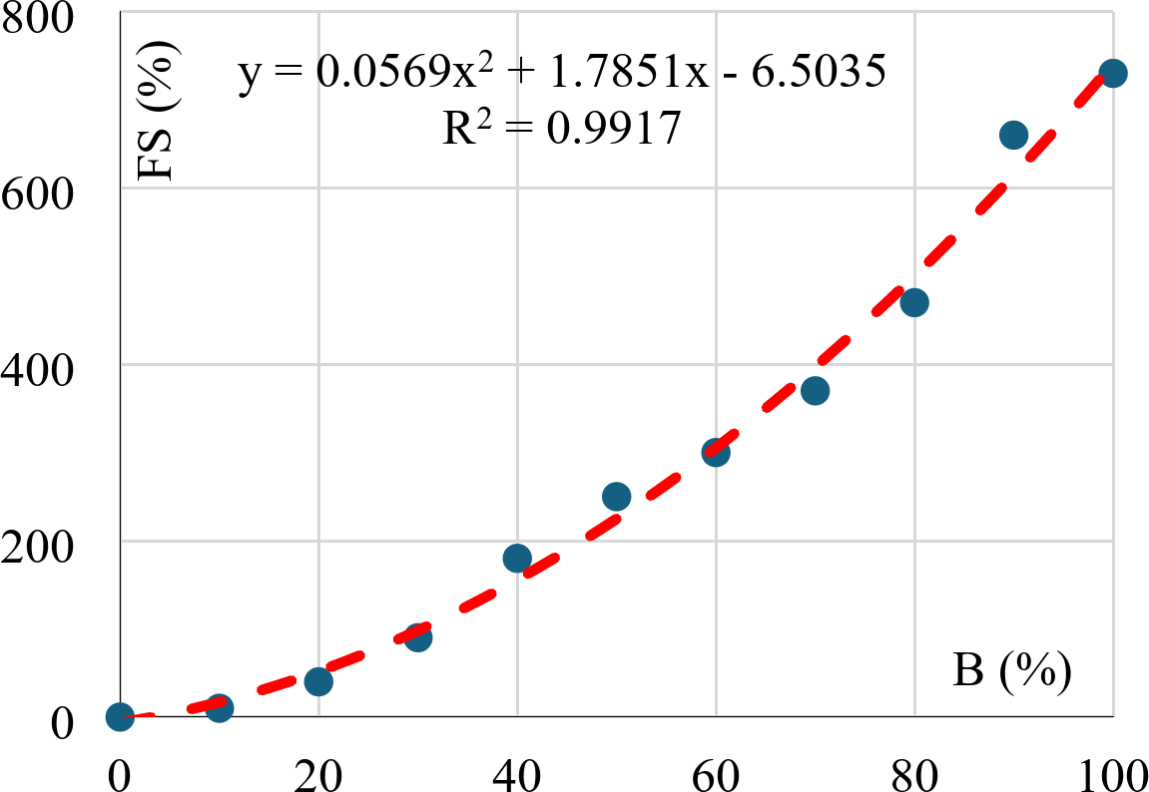
Free swell vs. bentonite content.

Finally, Fig. 15 illustrates the nonlinear relation between the free swell and the Bentonite content. This relation matches the (SSA Vs. B) that is shown in Fig. 8. As the Bentonite content increases, the SSA of the mixture increases, hence, the required amount of water to cover each particle increases which expands the mixture volume.

## Conclusions

This study aimed to investigate the influence of Bentonite content on the properties of Bentonite-Kaolinite mixtures, addressing gaps in the existing literature regarding their combined effect. Previous research has highlighted the impact of Bentonite on clay behavior, but limited studies have explored the interaction between Bentonite and Kaolinite in mixed systems. The study results could be concluded in as follows:

- As the Bentonite content increases, the specific unit weight and plasticity index show a clear upward trend, indicating that the mixture becomes more cohesive and capable of retaining water. This behavior is further evidenced by the grain distribution curve, which reveals an increase in specific surface area with more Bentonite, leading to higher water absorption and swelling potential, as reflected in the free swell index.
- The liquid limit, plastic limit, and plasticity index tests further emphasize how the addition of Bentonite increases the plasticity of the mixture, making it more moldable and moisture-retentive. Similarly, the optimum moisture content increases with increasing the bentonite content, it reaches the peak of 26% at a Bentonite content of 30% after it stabilized while the maximum dry density decreases due to the expansive nature of bentonite.
- Additionally, the activity index, which is determined from the plasticity index and clay content, effectively predicts the swelling and water absorption behavior of the mixtures. This index shows a strong correlation with the Bentonite content, further demonstrating how the swelling potential of the mixture increases with increasing the Bentonite content.
- The main innovation of this research is the extensive numerical correlations between the mixture ratio and mixture properties, which has not been extensively explored in prior studies. These numerical correlations provides a valuable tool to design mixtures with desired properties for many geotechnical applications such soil stabilization and barrier technologies.
- The results of this study are limited by the considered types of Bentonite and Kaolinite.

Future research should investigate the long-term behavior of these mixtures under different environmental conditions, which will enhance predictive models and optimize the practical application of Bentonite-Kaolinite mixtures in real-world projects. Additionally, further studies could explore more advanced parameters for Bentonite-Kaolinite mixtures, such as shear strength, consolidation properties, and both vertical and lateral swelling pressures.

## Data Availability

Data is provided within the manuscript in Table 2.
